# Diagnosis and treatment of malaria in peripheral health facilities in Uganda: findings from an area of low transmission in south-western Uganda

**DOI:** 10.1186/1475-2875-6-39

**Published:** 2007-04-02

**Authors:** Richard Ndyomugyenyi, Pascal Magnussen, Siân Clarke

**Affiliations:** 1 Vector Control, Division, Ministry of Health, P.O. Box 1661, Kampala, Uganda; 2 DBL Institute for Health Research and Development, Jaegersborg Allé ID 2920 Charlottenlund, Denmark; 3 Gates Malaria Partnership, Department of Infectious and Tropical Diseases, London School of Hygiene and Tropical Medicine, 50 Bedford Square, London WC1B 3DP, UK

## Abstract

**Background:**

Early recognition of symptoms and signs perceived as malaria are important for effective case management, as few laboratories are available at peripheral health facilities. The validity and reliability of clinical signs and symptoms used by health workers to diagnose malaria were assessed in an area of low transmission in south-western Uganda.

**Methods:**

The study had two components: 1) passive case detection where all patients attending the out patient clininc with a febrile illness were included and 2) a longitudinal active malaria case detection survey was conducted in selected villages. A malaria case was defined as any slide-confirmed parasitaemia in a person with an axillary temperature ≥ 37.5°C or a history of fever within the last 24 hrs and no signs suggestive of other diseases.

**Results:**

Cases of malaria were significantly more likely to report joint pains, headache, vomiting and abdominal pains. However, due to the low prevalence of malaria, the predictive values of these individual signs alone, or in combination, were poor. Only 24.8% of 1627 patients had malaria according to case definition and > 75% of patients were unnecessarily treated for malaria and few slide negative cases received alternative treatment.

**Conclusion:**

In low-transmission areas, more attention needs to be paid to differential diagnosis of febrile illnesses In view of suggested changes in anti-malarial drug policy, introducing costly artemisinin combination therapy accurate, rapid diagnostic tools are necessary to target treatment to people in need.

## Background

Effective case management (ECM) remains a cornerstone for reduction of malaria morbidity and mortality in sub-Saharan Africa [1]. However, ECM depends on early recognition of symptoms and signs, which are interpreted as a malaria episode and the clinical skills of a peripheral health care worker as there are often no resources for laboratory diagnosis in most malarious endemic areas [2,3]. Perceived fever is the sign most health workers use to diagnose clinical malaria. However, studies in areas of intense transmission have found reported fever or a history of fever to be an unreliable indicator of clinical malaria [4] Nevertheless, in areas of low or unstable transmission, clinical signs and symptoms might be more useful in diagnosing malaria amongst populations with low immunity. Although studies conducted in areas of low endemicity in Asia also found that none of the reported symptoms or signs was a good predictor of malaria [5,6], how symptoms are perceived is culturally determined and, therefore, the validity of potential diagnostic symptoms has to be tested in different socio-cultural and epidemiological settings [6].

Reliance on presumptive clinical diagnosis, in the absence of laboratory diagnosis, results in diagnostic inaccuracy, and over-diagnosis of malaria is common [2,7]. Higher rates of over-diagnosis are seen in areas of lower malaria transmission [7,8]. Uganda has uniform treatment guidelines which stipulate that any patient with fever without evidence of other diseases should treated for malaria even with a negative blood smear for malaria parasites and do not distinguish between the diagnosis of malaria in adults and children or in areas of low or high transmission. Improved diagnostic accuracy is essential to avoid wastage of drugs, and becomes of increasing importance with the higher drug costs associated with the introduction of artemisinin combination therapy (ACTs) [9]. Rapid malaria diagnostic tests offer a solution in situations where microscopy is limited and are increasingly being used in many malaria endemic areas [10-12] although there are wide variability in sensitivity both within and between products. The predictive value of diagnostic tests depends on the prevalence of infection, and the utility of rapid tests in diagnosing malaria will differ according to endemicity. A study was conducted to assess the diagnostic performance of signs and symptoms used by health workers to diagnose malaria and the performance of a rapid diagnostic test in comparison with microscopy in an area of low transmission in south-western Uganda.

## Materials and methods

### Study area

The study was conducted in Kamwezi sub-county in Kabale district, situated in south-western Uganda with a catchment population of about 9,300 people. The district lies at an altitude between 1,219 and 2,347 m above see level and covers an area of 1,827 km^2^. According to the 2002 census, the population of Kabale was 461,785 people with a population density of 290 persons per Km^2^. The average air temperature is 17.5°C, and occasionally drops to 10°C at night. The average rainfall is between 1,000 and 1,480 mm per annum with two peaks, March to June and September to December. Malaria transmission is low and unstable and people of all ages are at risk of malaria. The district is epidemic-prone, and occasional epidemic outbreaks occur shortly after peaks of rainfall [13]. Lying at an altitude between 1350 and 1900 above sea level, the Kamwezi study area is the most frequently affected by malaria epidemics and > 98% of the cases are caused by *Plasmodium falciparum*

## Methods

The study had two components: 1) passive case detection where all patients attending the out patient clinic with a febrile illness were included and 2) a longitudinal active malaria case detection survey was conducted in selected villages.

### Evaluation of clinical signs and symptoms in diagnosing malaria

All patients attending the outpatient clinic of a Health Centre IV in Kamwezi sub-county (a Health Centre IV has in- and out-patient facilities, an operating theatre and is staffed with a medical doctor) from December 2001 to March 2003 complaining of a febrile illness were included in the study. The history of illness, the presenting symptoms, their duration and axillary body temperature were recorded on a case record form developed for the study. Patients were asked about what diseases they perceived themselves to be suffering from based on the presenting symptoms. A full clinical examination of patients was undertaken by the health facility workers and was more comprehensive than routine examination, including examination for clinical anaemia and respiratory tract infection, and a presumptive diagnosis was made based on presenting signs and symptoms. Those patients suspected to be having malaria were treated according to national guidelines with a combination of chloroquine (CQ) and sulfadoxine-pyrimethamine (SP), which was by then the approved first line treatment and an *in vivo *study had shown that malaria parasites in the area were sensitive to this drug combination [14]. Patients with other diagnoses were treated accordingly. Haemoglobin (Hb) was measured using a portable HemoCue photometer (HemoCue^®^, Ängelholm, Sweden). Hb < 80 g/l was considered as severe anaemia, Hb 80–100 g/l as moderate anaemia and >100 g/l as normal. Hb was not adjusted for altitude. A thick blood film was also prepared for the detection of malaria parasites from all patients suspected of having malaria.

### Evaluation of a rapid diagnostic test in diagnosing malaria

A longitudinal active malaria case detection was conducted in four villages randomly selected by balloting from the list of the Health Centre catchment villages. The villages were categorized as far (at a distance >5 km) or near (within a distance < 5 km) from the health centre. Two villages were randomly selected in each category and they had a total population of about 800 people. The health workers examined all household members with fever or a history of fever within 24 hrs. The history of illness, presenting symptoms, and axillary body temperature were recorded on a case record form developed for the study. A sample of blood was collected from a finger prick using a dipstick capillary tube for antigen detection using the Uni-Gold™ Malaria (p.f) test (Uni-Gold™, Trinity Biotech, Wicklow, Ireland) based on detection of histidine-rich protein II (PfHRP-II). Health workers were trained to interpret the test results in the field and administer treatment. A thick blood film was also prepared for the detection of malaria parasites from all patients suspected of having malaria in the villages.

### Malaria case definition

All blood slides were stained and kept at the Health Centre and collected monthly by the principal investigator and examined by a senior laboratory technician at the Vector Control Division, Ministry of Health, Kampala. Another laboratory technician read at least a random 10% of the slides to ensure quality control. There were few discrepancies in the slide readings by the two technicians. In cases where there were discrepancies, the two technicians came together and re-examined the slides and made a collective decision on the reading. There were few patients who had only gametocytes but were not classified as malaria cases unless they had fever or a history of fever within the last 24 hrs and no symptoms suggestive of other febrile illness. Parasites were counted against 200 leukocytes and expressed as number of parasites/μl of blood assuming a standard leukocyte count of 8,000/μl. A malaria case was defined as any slide-confirmed parasitaemia in a person with an axillary temperature ≥ 37.5°C or a history of fever within the last 24 hrs and no signs suggestive of other diseases.

### Ethical considerations

Ethical approval for the study was obtained from the Uganda National Council for Science and Technology and the Danish National Committee on Biomedical Research Ethics. Permission to conduct the study was obtained from the district authorities. Participation in the study was voluntary after informed consent.

### Data analysis

Data were entered and analyzed using SPSS version 10.0. In order to obtain the best subset of symptoms for prediction of parasitaemia or a malaria episode, symptoms were evaluated using a nominal regression method based on the probability of likelihood ratios.

## Results

A total of 1627 patients, attending the Health Centre between December 2001 to March 2003 and given a presumptive clinical diagnosis of malaria, were interviewed (caretakers of children < 5 years responded for their child) about their presenting symptoms and what diseases they perceived themselves to be suffering from. Between 52 and 160 presumptive diagnoses of malaria were made each month based on history of fever, headache, joint pains, rigors and raised axillary body temperature (> 37.5°C). Mean age was 27 years (range 1–85 years) and 804 (49.4%) of patients were female. The majority of patients were adults (> = 16 years,1182) with 101 children less than five years and 344 patients aged 5–15 years. Most patients (1367, 84.0%) perceived themselves to be suffering from malaria, whilst 32 (2.0%) thought that they had malaria with a respiratory tract infection, and 215 (13.4%) were not sure what diseases they were suffering from, and for 13 (0.8%) no records were available.

**Figure 1:**
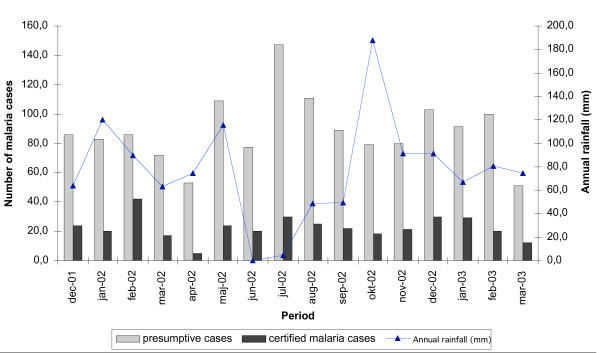
Relationship between presumptive and certified malaria cases and rainfall in Kabale district during the period December 2001 to March 2000.

A blood slide was prepared for 1577 (96.9%) of the patients suspected to be having malaria and 794 (50.3%) were found to be parasitaemic. Overall, 391 (24.8%) were classified as malaria cases according to the case definition (axillary temperature ≥ 37.5°C with positive blood slide confirmation): with 29.4%, 23.6% and 24.8% in the age groups < 5, 5–15 and ≥ 16 years respectively (table 1). The numbers of presumptive malaria cases were much higher than the certified cases for all months in the year. Although there was no marked seasonality, both presumptive and certified malaria cases tended to peak after the peaks of rainfall (Figure 1). As shown in Table 1, 435 (27.6%) patients had an elevated temperature without malaria parasitaemia. An elevated temperature in the absence of parasitaemia was highest amongst pre-school children, with 43.5%, 23.5% and 27.6% in the age groups < 5, 5–15 and ≥ 16 years respectively, (*P *= 0.001). Not all parasitaemic cases were febrile. Parasitaemia in the absence of demonstrable fever was found in 25.6% of patients overall, and was more common in older children and adults with 14.1%, 27.8% and 25.8% in the age groups < 5, 5–15 and 16 years respectively, (*P *= 0.03). Parasite density was generally low with only 25 % of the patients having parasite density ≥ 1,000/μl of blood.

**Table 1:** Fever and parasitaemia status by age group among patients presenting with a history of febrile illness at a peripheral health facility in South-western Uganda

Parasitaemia status	Fever	Age group in years			*P*-value
		
		< 5	5–15	≥ 16	
Parasitaemic	Febrile^1^	25 (29.4%)	77 (23.6%)	289 (24.8%)	*P *= 0.5
	Afebrile	12 (14.2%)	91 (27.8%)	300 (25.8%)	*P *= 0.03
Aparasitaemic	Febrile^1^	37 (43.5%)	77 (23.5%)	321 (27.6%)	*P *= 0.001
	Afebrile	11 (12.9%)	82 (25.1%)	255 (21.9%)	*P *= 0.05
Total (%)		85 (100%)	327 (100%)	1165 (100%)	

^1^Axillary temperature = 37.5°C

### Diagnostic performance of symptoms used by health workers to diagnose malaria

The symptoms most frequently reported by suspected malaria patients presenting to the health facility were headache (84%), fever (79%), joint pain (73%), loss of appetite (64%), vomiting (55%), and general weakness (55%). Parasitaemia status by reported symptoms among patients visiting the health facility is summarized in Table 2. For most of the reported symptoms, there was little difference in the frequency of symptoms according to parasitaemia status. Nominal regression (likelihood ratio) was used to determine which symptoms were associated with parasitaemia. Only headache, vomiting, and rigors were significantly associated with parasitaemia (*P *= 0.02, *P *= 0.02, and *P *= 0.03 respectively), whilst dizziness and cough had negative association with parasitaemia (*P *= 0.03, and *P *= 0.001 respectively). The overall sensitivity, specificity and positive predictive values of individual symptoms for predicting *Plasmodium falciparum *parasitaemia are also summarized in Table 2. Headache had the highest sensitivity (84.4%) followed by joint pains (78.1%), fever (77.2%) and loss of appetite (63.5%). However, the specificity of individual symptoms was 20.0%, 22.1%, 21.1% and 38.6% for headache, joint pains, fever, and loss of appetite, respectively. Most symptoms also had low positive predictive values for parasitaemia, typically ranging between 45 – 51%.

**Table 2:** Sensitivity and specificity of reported symptoms for diagnosing *Plasmodium falciparum *parasitaemia among patients attending a peripheral health facility in Kabale District, SW Uganda

Presenting symptoms	Parasitaemia Present (%): *n *= 750	Parasitaemia Absent (%): *n *= 856	*P- *value^1^	Sensitivity (%)	Specificity (%)	Positive predictive value (%)	Negative predictive value (%)
Headache	633 (84.4)	685 (80.0)	*P=*0.02	84.4	20.0	48.0	59.5
Joint pain	586 (78.1)	667 (77.9)	n.s.	78.1	22.1	46.8	53.5
History of fever	579 (77.2)	667 (77.9)	n.s.	77.2	21.1	46.5	52.5
Loss of appetite	476 (63.5)	526 (61.4)	n.s.	63.5	38.6	47.5	54.6
Vomiting	426 (56.8)	445 (52.0)	*P *= 0.02	56.8	48.0	48.9	55.9
General weakness	423 (56.4)	452 (52.8)	n.s.	56.4	47.2	48.3	55.3
Rigors	138 (18.4)	133 (15.5)	*P *= 0.03	18.4	84.5	50.9	54.2
Cough	124 (16.5)	195 (22.8)	*P *= 0.001	16.5	77.2	38.9	51.4
Dizziness	76 (10.1)	112 (13.1)	*P *= 0.03	10.1	86.9	40.4	52.5
Abdominal pain	73 (9.7)	75 (8.8)	n.s.	9.7	91.2	49.3	53.6
Backache	35 (4.7)	42 (4.9)	n.s.	4.7	95.1	45.5	52.6
Flu-like symptoms	28 (3.7)	30 (3.5)	n.s.	3.7	96.5	48.3	53.4

^1 ^χ^2 ^values calculated using log likelihood, df = 1; n.s. not significant

Reported symptoms among health facility patients fulfilling the case definition of malaria, and non-malaria cases are summarized in table 3. For many of the symptoms, there was little difference in the frequency of symptoms reported by malaria cases and non-cases. Headache, vomiting and abdominal pain were significantly associated with being a malaria case (*P *= 0.009, *P *= 0.03 and *P *= 0.03 respectively), while patients reporting joint pains (*P *= 0.009) were less likely to be a malaria case. For diagnosis of a malaria case, headache had the highest sensitivity (84.7%), followed by joint pains (73.2%), fever (74.7%), loss of appetite (61%) and vomiting (59.2 %). However the specificity of each symptom was low 19.0%, 20.2%, 21.1 %, 37% and 47.9%, for headache, joint pains, fever, loss of appetite and vomiting, respectively. Further analysis was done to identify a combination of symptoms with a high sensitivity and specificity predictive of a malaria episode. However, no clinical algorithm could be identified.

**Table 3:** Sensitivity and specificity of reported symptoms for diagnosing malaria^1 ^among patients attending a peripheral health facility in Kabale District, SW Uganda.

Presenting symptoms	Malaria case^1 ^(%): *n *= 392	Not malaria case (%): *n *= 1187	*P- *value^2^	Sensitivity (%)	Specificity (%)	Positive predictive value (%)	Negative predictive value (%)
Headache	332 (84.7)	962 (81.0)	*P *= 0.009	84.7	19.0	25.7	21.1
Joint pain	287 (73.2)	947 (79.8)	*P *= 0.009	73.2	20.2	23.3	30.4
History of fever	293 (74.7)	936 (78.9)	n.s.	74.7	21.1	23.8	28.3
Loss of appetite	239 (61.0)	747 (62.9)	n.s.	61.0	37.0	24.3	25.8
Vomiting	232 (59.2)	619 (52.1)	*P *= 0.03	59.2	47.9	27.3	22.0
General weakness	216 (55.1)	648 (54.6)	n.s.	55.1	45.4	25.0	24.6
Rigors	76 (19.4)	190 (16.0)	n.s.	19.4	84.0	28.6	24.1
Cough	75 (19.1)	236 (19.9)	n.s.	19.1	80.1	24.1	25.0
Dizziness	48 (12.2)	137 (11.5)	n.s.	12.2	88.5	25.9	24.7
Abdominal pain	50 (12.8)	96 (8.1)	*P *= 0.03	12.8	91.9	34.2	23.9
Backache	17 (4.3)	58 (4.9)	n.s.	4.3	95.1	22.7	24.9
Flu-like symptoms	17 (4.3)	40 (3.4)	n.s.	4.3	96.6	29.8	24.6

^1^Malaria case defined as: parasitaemia and axillary temperature ≥ 37.5°C

^2 ^χ^2 ^values calculated using log likelihood, df = 1; n.s. not significant

A total of 1,516 patients had their Hb measured (79, 317 and 1120 in the age groups < 5, 5–15 and ≥ 16 years, respectively). Moderate anaemia and severe anaemia was found in 6.7% and 2.4% of patients, respectively. Anaemia was found in 57.0%, 88% and 94% of patients in the age groups < 5, 5–15 and ≥ 16 years, respectively (*P *< 0.001). Severe anaemia was found in 16%, 3% and 1% of patients in the age groups < 5, 5–15 and >16 years, respectively (*P *< 0.001). Hb level was not associated with parasitaemia or being a malaria case.

### The performance of the rapid diagnostic test (RDT) in comparison with microscopy

During active case detection 669 patients had a blood slide prepared and RDT done. Of these 371 (55.5%) were smear-positive and 391 (58.4%) were RDT positive. Of the RDT positive samples, 243 (65.5%) were also smear-positive, and 148 (49.7%) were smear-negative (table 4). One hundred twenty eight samples were RDT negative but smear-positive (34.5%). Considering microscopy as the gold standard, the sensitivity and specificity of RDT were 64.6% and 50.3%, respectively. The positive and negative predictive values were 64.6% and 50.3 %, respectively. A comparison was also made on the intensity of infection and positivity of the RDT, which was highest for high intensity parasitaemia, detecting 64.5% of parasitaemia ≥ 500 parasites/μL, with 35.5% high density parasitaemia remaining undetected (Table 5).

**Table 4:** Performance of rapid diagnostic test in comparison to microscopyin identifying parasitaemia.

Rapid diagnostic test	Parasitaemia on microscopy		Total
		
	Positive	Negative	
Positive	243 (65.5 %)	148(49.7%)	391
Negative	128(34.0 %)	150(50.3%)	278
Total	371	298	669

**Table 5:** The results of the rapid diagnostic test categorized by the level of parasitaemia as determined by microscopy

Parasitaemia (asexual parasites/μL)	Results of rapid diagnostic test		Total
		
	No. of positive (%)	No. of negative (%)	
0	148(49.7)	150 (50.3)	298
1–99	55 (88.7)	7(11.3)	62
100–199	33(55.0)	27 (45.0)	60
200–499	57(58.8)	40 (41.2)	97

≥ 500	98(64.5)	54(35.5)	152

^1^Malaria case defined as: parasitaemia and axillary temperature ≥ 37.5°C ^2 ^χ^2 ^values calculated using log likelihood, df = 1; n.s. not significant

### Malaria diagnosis and prescription practice at the health facility

A presumptive diagnosis of malaria was made on 1,627 patients but only 24.8% had a malaria episode according to the case definition. A presumptive diagnosis of malaria was given to 1,224 (75.2%) of the patients, 3.4% were diagnosed as malaria plus anaemia, 20% as malaria with respiratory tract infection and 1.4% as malaria and a chronic disease. Of the 1627 patients suspected to be having malaria, 54.3% were treated with a combination of SP and CQ (47.8% with CQ +SP and 6.8% with CQ + SP + an antibiotic) in accordance with National policy. However, about 34% of the patients were treated with CQ without SP (22% with CQ alone and 11.5% with CQ+ an antibiotic) and 8.7% with SP only in combination with haematinics or antibiotics. All cases were treated with at least one antimalarial and 22% were also treated with an antibiotic. Among the 783 cases who were slide negative only 215 (27.5%) received antibiotic treatment (mainly co-trimoxazole and penicillin V tablets)

## Discussion

In malaria holoendemic areas with no laboratory facilities, anti-malarial treatment is recommended for all patients with fever or a history of fever [15]. However, although fever is the characteristic sign of clinical malaria, many *P. falciparum *infections in endemic areas do not present with elevated temperature [16]. Studies in areas of high transmission have shown fever or a history of fever with asexual parasitaemia of any density, to have a sensitivity and specificity of 70.4% and 68.9%, respectively for diagnosis of malaria [2]. Slide confirmation is also of limited utility in areas of intense transmission, as asymptomatic carriage of malaria parasites occurs frequently due to high tolerance to malaria parasites [17,18] and the detection of parasites in a blood film or antigens from afebrile individuals does not necessarily indicate clinical malaria [16]. However the pattern of clinical malaria varies with the intensity of transmission, and in areas of low transmission, any *P. falciparum *infection may be synonymous with a malaria episode due to lack of acquired immunity [19,20] and the sensitivity of diagnostic approaches may differ. In Thailand, an area of low transmission, a history of fever and headache without cough was found to have a sensitivity of 51% and a specificity of 72% for diagnosis of malaria among 1–15 year old children [5]. However, other studies have shown that none of the reported symptoms were good predictors of malaria in an area of low malaria transmission in India [6]. In the present study history of fever was commonly reported but was an unreliable indicator being neither associated with presence of parasitaemia nor with fulfilling the malaria case definition. Headache had the highest sensitivity (84.4%), followed by vomiting (59.2%), for diagnosis of clinical malaria. However, the specificity and positive predictive value of clinical signs and symptoms were generally low, and a reliable clinical algorithm appropriate for the diagnosis of malaria in a low transmission area could not be formulated. This could partly be due to restrictive case definition used in this study as patients with parasitaemia who were not febrile were not classified as malaria cases. This case definition was used because earlier studies in the area showed that only about 60% of asymptomatic parasitaemic individuals became symptomatic within 14 days of follow-up (unpublished data), indicating that probably malaria endemicity in this area could have changed from hypo to meso-endemic due to several factors including climatic changes. The predictive value of diagnostic tests and clinical algorithms is strongly dependent on disease prevalence, and the poor predictive values of individual symptoms observed in this study area reflects the low prevalence of parasitaemia and malaria in highland areas. In addition, fever or *"Omushwija" *(the local term for fever in the study area) is a broad term meaning any febrile illness, which could include acute respiratory tract infections that were also common and the reason why more than 27.6 % of the patients had elevated temperature without parasitaemia. Similarly, other symptoms such as headache, rigors and joint pains may occur in other febrile illness making them non-specific for malaria. Severe anaemia was most frequently seen in children less than five years as previously observed [21]. However, in our study Hb level was associated neither with parasitaemia nor with a malaria episode, indicating that malaria is not an important cause of anaemia in areas of low transmission as opposed to areas of high transmission [23].

A presumptive diagnosis of malaria was made on 1627 patients but only 24.8% had a malaria episode according to the case definition, resulting in more than 75% of the patients receiving anti-malarial drugs unnecessarily. Only about 18% of the patients received antibiotics. Over diagnosis of malaria in sub-Saharan Africa is common [2,8]. By treating all febrile cases as malaria leads to over diagnosis of malaria and this may cause other infections to be under diagnosed and untreated leading to high morbidity and mortality. The presented data confirm the findings of others, and show that even in a highland area of low malaria transmission insufficient thought is given to alternative causes for fever. The overuse of anti-malarial treatments could be due to inadequate skills of peripheral health workers in diagnosing malaria, unreliability of signs and reported symptoms in making the diagnosis of malaria (as the present data suggest) and lack of a standard case definition for malaria in areas of low transmission. In view of the emerging resistance to the commonly available anti-malarial drugs and the recommended change in anti-malarial drug policy to introduce the more costly artemisinin combination therapy (ACT), identification and treatment of only malaria cases with antimalarials would prevent drug misuse and development of drug resistance. Information on the cost-effectiveness of various diagnostic tools and the treatment regimens putting into consideration diagnostic accuracy such as reductions in illness, death and drug resistance is needed to reduce over-diagnosis of malaria and misuse of more expensive drugs [9]. Lack of expertise, maintenance of microscopes reagents, delays in results and inadequate quality control at peripheral health care facilities make microscopical diagnosis problematic [3,23] and RDTs are increasingly being used as alternative to microscopy in many malaria endemic areas [10-12].The sensitivity of 65.5 % of the RDT used in this study was lower than what has been previously observed [10], which could be due to unreliability of microscopy and RDTs especially in areas of low prevalence and parasite density like in this study area. We can not rule out the batch effect or storage conditions prior to purchase which could have compromised the reliability of the results Yet to improve diagnosis and treatment in populations with little or no immunity, a high-sensitivity diagnostic test is vital to ensure that malaria-infected patients do not go untreated.

The interpretation of the RDT by health workers during case detection could not have been influenced by haemoglobin level since it was not measured at community level or by blood slide readings which was done at later stage in Kampala. The health workers used presumptive diagnosis to treat all patients suspected to be having malaria with the recommended anti-malarial drugs in the national guidelines. The sensitivity of RDT could have been higher than what is observed in this study if more sensitive diagnostic tests such as polymerase chain reaction (PCR) had been used as the gold standard.

In the absence of more reliable malaria diagnosis, the current practice of treating all febrile infections with antimalarial drugs remains unavoidable in low transmission areas, despite increasing drug costs. Although RDTs are easy to use, require minimal expertise and no special equipment, the variability in sensitivity both within and between products calls for an urgent development of high quality, accurate, rapid and affordable diagnostic tools for malaria so that new anti-malarial drugs which are costly and associated with increased toxicity are targeted to people with definite malaria illness.

## Conflicts of interest statement

The author(s) declare that they have no competing interests.

## Authors' contributions

The authors, roles in the research process and in the preparation of the manuscript were as follows:

RN, PM & SC participated in the conception and design of the study. RN implemented the study and collected data. RN performed the data and statistical analysis and wrote the manuscript. PM and SC supervised field work. All authors read and approved the final manuscript:
